# Log odds of positive lymph nodes compared to positive lymph node ratio and number of positive lymph nodes in prognostic modeling for patients with NSCLC undergoing lobectomy or total pneumonectomy: a population-based study using Cox regression and XGBoost with SHAP analysis

**DOI:** 10.3389/fsurg.2024.1530250

**Published:** 2025-01-20

**Authors:** Qiming Huang, Shai Chen, Zhenjie Li, Longren Wu, Dongliang Yu, Linmin Xiong

**Affiliations:** ^1^ Department of Cardiac Surgery, The Second Affiliated Hospital, Jiangxi Medical College, Nanchang University, Nanchang, Jiangxi, China; ^2^ Department of Vascular Surgery, The Second Affiliated Hospital, Jiangxi Medical College, Nanchang University, Nanchang, Jiangxi, China; ^3^ Department of Thoracic Surgery, The Second Affiliated Hospital, Jiangxi Medical College, Nanchang University, Nanchang, Jiangxi, China

**Keywords:** log odds of positive lymph nodes, positive lymph node ratio, number of positive lymph nodes, NSCLC, SEER

## Abstract

**Background:**

Methods such as the number of positive lymph nodes (nPLN), lymph node ratio (LNR), and log odds of positive lymph nodes (LODDS) are used to predict prognosis in patients with non-small cell lung cancer (NSCLC). We hypothesized that LODDS could be a superior independent predictor of prognosis and aimed to compare its effectiveness with nPLN and LNR in predicting survival outcomes in stage I-IIIA NSCLC patients.

**Methods:**

We utilized data from the Surveillance, Epidemiology, and End Results (SEER) 17 registry (2010–2019) to study NSCLC patients, focusing on those who underwent surgery with confirmed lymph node involvement (N1 or N2 disease). We aimed to compare overall survival (OS) and cancer-specific survival (CSS) based on nPLN, LNR, and LODDS. Kaplan-Meier and Cox regression analyses were employed to evaluate survival, with thresholds determined using X-tile software. An XGBoost model was constructed to predict overall survival in patients using three features: LODDS, LNR, and PLN. SHapley Additive exPlanations (SHAP) analysis was applied to assess feature importance and provide interpretable insights into the model's predictions.

**Results:**

The study analyzed 3,132 eligible NSCLC patients from the SEER database, predominantly male (53.07%) with adenocarcinoma (43.65%) or squamous cell carcinoma (29.76%). Survival outcomes were assessed using nPLN, LNR, and LODDS. LODDS showed superior predictive value for both OS and CSS compared to nPLN and LNR, as indicated by a larger Log Likelihood Ratio (LLR) and smaller Akaike Information Criterion (AIC). Higher scores on npLN, LNR, and LODDS were strongly related with a poorer prognosis, according to Kaplan-Meier analyses (*P* < 0.001). The SHAP (SHapley Additive exPlanations) analysis of the XGBoost model demonstrated that the LODDS exhibited the highest SHAP values (0.25) for predicting overall survival in patients, consistently outperforming the LNR and the number of nPLN across both training and validation datasets.

**Conclusions:**

Compared to the nPLN and LNR staging systems, LODDS demonstrates superior prognostic power for patients with stage I–IIIA NSCLC undergoing lobectomy or pneumonectomy. By integrating both positive and negative lymph node information, LODDS offers a refined risk stratification that is particularly valuable in cases with high lymph node heterogeneity.

## 1 Introduction

Lung cancer is the most prevalent cancer in China and the second most common in the United States, with approximately 85% being non-small cell lung cancer (NSCLC) (1). Accurate stratification of NSCLC patients based on survival outcomes is crucial for effective treatment. The American Joint Committee on Cancer Tumor Lymph Node Metastasis (TNM) Lung Cancer Staging System classifies lymph node staging based on the anatomical location of the lymph nodes involved, disregarding the absolute number of lymph nodes affected. Moreover, it does not specify the number of lymph nodes or stations examined but recommends sampling at least 6–10 lymph nodes or stations (2, 3). Ludwig et al. demonstrated that postoperative survival in NSCLC patients correlates with the number of lymph nodes resected during surgery, suggesting that the optimal number is 11 (3).

In an analysis of 38,806 NSCLC cases from the SEER registry and 5,706 NSCLC cases from a Chinese registry, Liang et al. found that a higher number of examined lymph nodes was associated with more accurate lymph node staging and improved long-term survival in resected NSCLC cases, recommending 16 examined lymph nodes as a threshold for postoperative prognostic stratification in patients deemed lymph node-negative (4).

Additionally, other methods of lymph node assessment have been shown to better predict prognosis in lymph node-positive NSCLC patients (5–10). Emerging evidence suggests that the number of histologically positive lymph nodes and the lymph node ratio have prognostic significance across various cancers, including esophageal, thyroid, breast, peripancreatic, gastric, colorectal, and cervical cancers, with similar findings in NSCLC studies (11–17). The nPLN and LNR have been identified as independent predictors of survival post-NSCLC resection. However, the superiority of one method over the other in NSCLC remains unclear. Another ratio-based approach, the LODDS, is currently employed as a prognostic indicator for various malignancies in several countries (10, 18–23). When a single lymph node is examined, the log odds of positive lymph nodes (LODDS) is calculated as the natural logarithm of the ratio between the probability of a positive lymph node and that of a negative lymph node. A preliminary report indicated that LODDS outperforms the number of positive lymph nodes (nPLNs) and pathological N-staging in predicting outcomes for stage I-IIIa NSCLC (24). However, this study did not address the relationship between LODDS and cancer-specific survival (CSS) nor analyze lymph nodes within stratified subgroups based on the number of nodes examined.

Thus far, no population-based study has elucidated the prognostic significance of LODDS for NSCLC. We posited that LODDS serves as an independent prognostic factor for these patients. To validate this hypothesis, we evaluated the comparative efficacy of LODDS, nPLNs, and the lymph node ratio (LNR) in predicting overall survival (OS) and CSS among lymph node-positive stage I-IIIA NSCLC patients who underwent lobectomy or total lung resection. We also explored the associations between these variables and survival outcomes.

## 2 Materials and methods

### 2.1 Data source and patient selection

We extracted data from the Surveillance, Epidemiology, and End Results Program (SEER) 17 registry study database, encompassing the most recent data available from 2010 to 2019. The SEER 17 database includes registries across various urban and rural regions of the United States. Cancer data collection initiates with the identification of cancer patients diagnosed or treated in hospitals, outpatient clinics, radiology departments, physician offices, laboratories, surgery centers, or other healthcare providers such as pharmacists. All 50 states mandate that newly diagnosed cancers be reported to a central registry. Cancer registries then review these reported cases to ascertain if the information is legally reportable. If deemed reportable, the registry extracts cancer information from the medical records as stipulated by the North American Association of Central Cancer Registries (NAACCR) Data Standards External Site Policy.

Initially, we identified patients aged 18 or older using center codes C34.0-C34.9, which correspond to lung and bronchus diagnoses. Subsequently, we refined our inclusion criteria to encompass individuals diagnosed with NSCLC between January 1, 2010, and December 31, 2019. We focused on patients who underwent primary site-specific surgery and presented with N1 or N2 disease, with histological confirmation of at least one lymph node. The histological tumor types were restricted to those specified in the International Classification of Diseases for Oncology, Third Edition, including squamous cell carcinoma, adenocarcinoma, and other variants. Inclusion criteria also mandated a minimum survival period of one month post-surgery and active follow-up. We extracted clinicopathological data such as age at diagnosis, N stage, histology, gender, race, surgical approach, grade, T stage and primary site.

Exclusion criteria encompassed the presence of multiple primary tumors, unexamined lymph nodes, unknown nPLN, distant metastases, and IIIB or IV stage. The study protocol was reviewed and approved by the Ethics Committee of the Second Hospital of Nanchang University, ensuring adherence to ethical standards and regulations.

### 2.2 Statistical methods

OS and CSS were the primary endpoints of this study. Correlations among the LODDS, LNR, and the nPLN were examined through smoothed fitted curves. The Kaplan-Meier method was employed to estimate OS and CSS, with the log-rank test used for comparative analysis of the survival estimates. X-tile software facilitated the determination of optimal thresholds for LNR and nPLNs. The X-tile software analyzes different thresholds using Kaplan-Meier survival curves to determine the optimal cutoff values for LNR and nPLNs. The software selects thresholds that maximize the differentiation of patient prognosis based on survival rate comparisons, ensuring clinical relevance. Specifically, the output heatmap visualizes the impact of various thresholds on survival, aiding researchers in making informed decisions. For the analysis of the number of positive lymph nodes (nPLN), patients were stratified into two groups using x-tile software (Supplementary Figure S1): npLN ≤ 2 and nPLN > 2. Similarly, for the LNR analysis, patients were classified into LNR ≤ 0.5 and LNR > 0.5.

Cox proportional hazards models were utilized to evaluate the impact of demographic, pathological, and treatment variables in both univariate and multivariate contexts. Stratified analysis was used to evaluate the impact of LNR, nPLN, and LODDS on survival across different histologic types, T stages, and N stages. The goodness of fit for the models was assessed using the Akaike Information Criterion (AIC) and the Log Likelihood Ratio (LLR), with lower AIC or higher LLR values signifying superior model fit.

The LODDS were calculated as the logarithm of the ratio of the probability of a positive lymph node to that of a negative lymph node, according to the formula: $\mathrm{LODDS}=\frac{\mathrm{positive}\;\mathrm{lymph}\;\mathrm{nodes}+0.5}{\mathrm{total}\;\mathrm{lymph}\;\mathrm{nodes}-\mathrm{positive}\;\mathrm{lymph}\;\mathrm{nodes}+0.5}$. The addition of 0.5 to both the numerator and the denominator was employed to avoid computational singularities. For LODDS analysis, patients were categorized into LODDS < 0.26 and LODDS ≥ 0.26. The values of LODDs were identified by recursive partitioning. Additionally, the number of lymph nodes examined was classified into <10 or ≥10, as the removal of 10 or more lymph nodes was associated with the longest median survival.

A predictive model for OS was constructed using the eXtreme Gradient Boosting (XGBoost) algorithm. The dataset was randomly divided into a training set (2,514 patients) and a validation set (618 patients). The primary endpoint was OS, with censoring applied to patients alive at the end of follow-up. Three lymph node-related features were selected based on their prognostic relevance in NSCLC: the LODDS, LNR, and nPLN. Model training was conducted on the training set, with hyperparameters optimized using five-fold cross-validation, and performance was evaluated on the independent validation set. SHAP (SHapley Additive exPlanations) values were calculated to quantify the contribution of each feature to the model's output, providing global rankings of feature importance as well as local interpretations for individual predictions. The predictive capacity of LODDS, LNR, and PLN was assessed by comparing their SHAP value distributions and mean SHAP importance scores. Statistical significance of differences in feature contributions was evaluated using non-parametric tests. All analyses were performed using Python (version 3.10), with key libraries including XGBoost, SHAP, and Scikit-learn. A *p*-value < 0.05 was considered statistically significant.

## 3 Results

### 3.1 Baseline characteristics of study participants

A total of 3,132 eligible patients meeting the criteria were identified from the SEER database. Patient demographics and tumor characteristics are detailed in Table 1. Of these patients, the majority were male (1,662, 53.07%), and the predominant histological types were adenocarcinoma (43.65%) and squamous cell carcinoma (29.76%). Regarding surgical procedures, approximately 87.71% underwent lobectomy, while 12.29% received total pneumonectomy. The mean number of lymph nodes resected was 13.89 (±SD: 9.75), with a median nPLN of 1. The median LNR was 0.14 (Q1-Q3: 0.08–0.27), and the median LODDS was −0.65 (Q1-Q3: −0.89 to −0.37) (Table 1).

**Table 1:** Baseline characteristics of the population.

	Median (Q1-Q3)
ELN	12.00 (7.00–18.00)
nPLN	1.00 (1.00–3.00)
LODDS	−0.65 (−0.89–0.37)
LNR	0.14 (0.08–0.27)
	N (%)
Age	
<65 years	894 (28.54%)
≥65 years	2,238 (71.46%)
Sex	
Male	1,662 (53.07%)
Female	1,470 (46.93%)
Race	
White	2,620 (83.65%)
Black	242 (7.73%)
Other	270 (8.62%)
Year_of_diagnosis	
2010–2014	1,606 (51.28%)
2015–2019	1,526 (48.72%)
Primary_site	
Upper lobe	1,616 (51.60%)
Middle lobe	171 (5.46%)
Lower lobe	1,151 (36.75%)
Main bronchus	53 (1.69%)
Other	141 (4.50%)
Grade	
I	395 (12.61%)
II	1,351 (43.14%)
III	1,331 (42.50%)
IV	55 (1.76%)
Histology	
SCC	932 (29.76%)
ADC	1,367 (43.65%)
ADSC	833 (26.60%)
T_stage	
T1	904 (28.86%)
T2	1,556 (49.68%)
T3	513 (16.38%)
T4	159 (5.08%)
N_stage	
N1	2,784 (88.89%)
N2	348 (11.11%)
Operation type	
Lobectomy	2,747 (87.71%)
Total pneumonectomy	385 (12.29%)

Note: Q1-Q3, interquartile range; SCC, squamous cell carcinoma; ADC, adenocarcinoma; ELN, examined lymph node; nPLN, number of positive lymph nodes; LNR, positive lymph node rate; LODDS, log odds of positive lymph nodes.

### 3.2 Association of LODDS, number of positive lymph nodes, and lymph node ratio with survival outcomes

**Figure 1:**
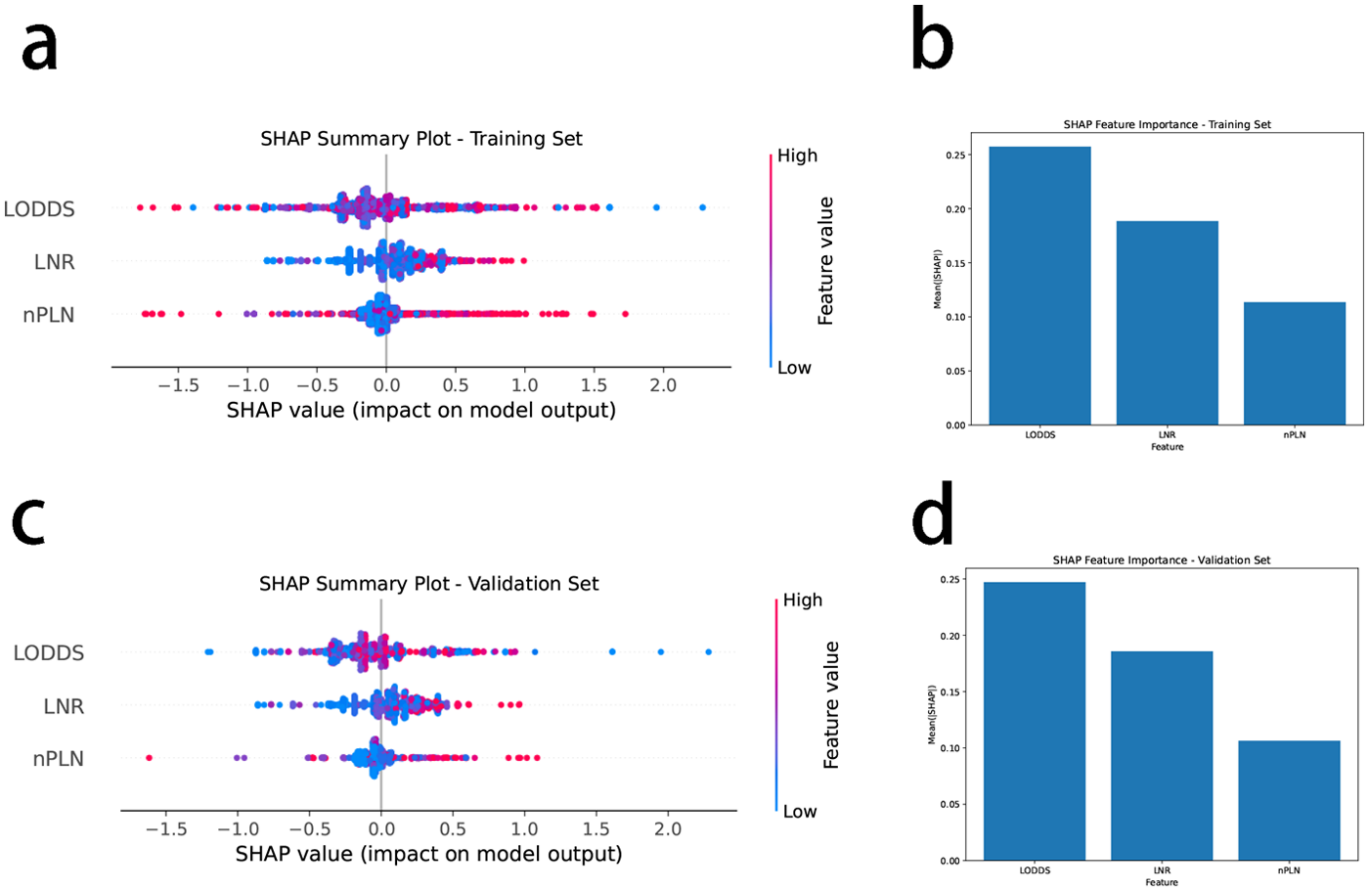
Smooth fitting curves for LODDS and LNR, nPLN. **(a)** LODDS and PLN: The red curve illustrates the relationship between LODDS (log odds of positive lymph nodes) and PLN (number of positive lymph nodes). As PLN increases, LODDS also rises; however, when PLN reaches around 22, the increase in LODDS plateaus. This suggests that beyond a high number of positive lymph nodes, additional PLN may have limited predictive value for survival. The blue dotted lines represent the confidence interval. **(b)** LODDS and LNR: The red curve shows the relationship between LODDS and LNR (lymph node ratio). As LNR increases, LODDS also rises, demonstrating a linear trend. The black tick marks along the *x*-axis indicate the distribution of LNR among patients, illustrating the range of LNR values in the sample.

Figure 1 illustrates the relationships among the nPLN, LNR, and LODDS. Notably, when the number of positive lymph nodes reaches 22, LODDS ceases to increase with the number of positive lymph nodes, whereas LODDS continues to rise in conjunction with LNR.

The univariate Cox regression analysis revealed that age, gender, histology, primary site, grade, histologic type, T stage, and surgical approach were significantly relatived with both OS and CSS (Table 2).

**Table 2:** Univariate analyses of cohort.

	Statistics	All-cause mortality HR (95%CI) *P*-value	Lung cancer-specific mortality rates HR (95%CI) *P*-value
Age			
<65 years	894 (28.54%)	1	1
≥65 years	2,238 (71.46%)	1.61 (1.43, 1.80) < 0.0001	1.56 (1.36, 1.78) < 0.0001
Sex			
Male	1,662 (53.07%)	1	1
Female	1,470 (46.93%)	0.68 (0.62, 0.75) < 0.0001	0.73 (0.66, 0.82) < 0.0001
Race			
White	2,620 (83.65%)	1	1
Black	242 (7.73%)	0.89 (0.74, 1.07) 0.2051	0.91 (0.73, 1.13) 0.3824
Other	270 (8.62%)	0.90 (0.75, 1.08) 0.2530	0.95 (0.77, 1.17) 0.6344
Primary_site			
Upper lobe	1,616 (51.60%)	1	1
Middle lobe	171 (5.46%)	0.75 (0.59, 0.95) 0.0153	0.66 (0.49, 0.88) 0.0051
Lower lobe	1,151 (36.75%)	1.00 (0.90, 1.11) 0.9652	1.00 (0.88, 1.13) 0.9861
Main bronchus	53 (1.69%)	1.06 (0.73, 1.53) 0.7742	1.16 (0.77, 1.76) 0.4788
Other	141 (4.50%)	1.25 (1.00, 1.55) 0.0469	1.28 (1.00, 1.65) 0.0543
Grade			
I	395 (12.61%)	1	1
II	1,351 (43.14%)	2.89 (2.33, 3.58) < 0.0001	2.80 (2.16, 3.63) < 0.0001
III	1,331 (42.50%)	3.70 (2.99, 4.58) < 0.0001	4.09 (3.16, 5.29) < 0.0001
IV	55 (1.76%)	3.48 (2.38, 5.09) < 0.0001	4.41 (2.89, 6.75) < 0.0001
Histology			
SCC	932 (29.76%)	1	1
ADC	1,367 (43.65%)	0.84 (0.76, 0.94) 0.0016	0.92 (0.80, 1.04) 0.1809
ADSC	833 (26.60%)	0.56 (0.49, 0.64) < 0.0001	0.65 (0.55, 0.75) < 0.0001
T_stage			
T1	904 (28.86%)	1	1
T2	1,556 (49.68%)	1.41 (1.25, 1.59) < 0.0001	1.42 (1.23, 1.64) < 0.0001
T3	513 (16.38%)	2.14 (1.85, 2.47) < 0.0001	2.22 (1.87, 2.64) < 0.0001
T4	159 (5.08%)	2.37 (1.91, 2.95) < 0.0001	2.49 (1.94, 3.21) < 0.0001
N_stage			
N1	2,784 (88.89%)	1	1
N2	348 (11.11%)	1.04 (0.87, 1.25) 0.6316	1.02 (0.83, 1.26) 0.8508
nPLN	2.59 ± 6.57	1.01 (1.00, 1.01) 0.0578	1.01 (1.00, 1.01) 0.0883
LODDS	−0.60 ± 0.45	1.33 (1.20, 1.48) < 0.0001	1.37 (1.22, 1.55) < 0.0001
LNR	0.27 ± 1.06	1.03 (1.00, 1.06) 0.0720	1.01 (0.96, 1.07) 0.6552
Operation type			
Lobectomy	2,747 (87.71%)	1	1
Total pneumonectomy	385 (12.29%)	1.66 (1.46,1.89) < 0.0001	1.62 (1.39,1.90) < 0.0001

Note: SCC, squamous cell carcinoma; ADC, adenocarcinoma; ELN, examined lymph node; nPLN, number of positive lymph nodes; LNR, positive lymph node rate; LODDS, log odds of positive lymph nodes.

Following these findings, three distinct multivariate models were developed to assess the predictive capacity of npLN, LNR, and LODDS. In the fully adjusted model, using OS as the outcome measure for the entire cohort, LNR and LODDS as continuous variables demonstrated predictive value for both OS and CSS (*P* < 0.05), whereas npLN did not show predictive significance (*P* = 0.0924). In the fully adjusted model, each unit increase in LODDS was associated with a 44% higher risk of overall mortality (OS) (HR = 1.44, 95% CI: 1.29–1.60, *p* < 0.0001), while increases in LNR and PLN were linked to 4% (HR = 1.04, 95% CI: 1.01–1.07, *p* = 0.0180) and 1% (HR = 1.01, 95% CI: 1.00–1.01, *p* = 0.0924) increases in risk, respectively. For cancer-specific survival (CSS), each additional unit of LODDS corresponded to a 45% higher risk of cancer-specific mortality (HR = 1.45, 95% CI: 1.28–1.64, *p* < 0.0001), while changes in LNR and PLN were associated with modest increases of 2% (HR = 1.02, 95% CI: 0.98–1.07, *p* = 0.3352) and 1% (HR = 1.01, 95% CI: 1.00–1.01, *p* = 0.1310) in risk, respectively (Table 3). Significant survival differences were observed across different groups. For patients with PLN ≤ 2, the 1-year, 3-year, and 5-year survival rates were 72.19%, 40.79%, and 21.53%, respectively, with a median survival time of 27 months. In contrast, patients with PLN > 2 had reduced survival rates of 59.42% at 1 year and 11.76% at 5 years, with a shorter median survival time of 17 months. Similar trends were observed for LNR and LODDS groups, with patients in the LNR ≤ 0.5 and LODDS ≤ 0.26 categories showing higher survival rates and longer median survival times. Overall, lower PLN, LNR, and LODDS values were associated with better survival outcomes. When CSS was used as the outcome, LODDS as a continuous variable was a significant predictor of both OS and CSS (*P* < 0.0001), while LNR (*P* = 0.3352) and nPLN (*P* = 0.1310) did not demonstrate predictive capability. The respective HR and CI are detailed in Table 3.

**Table 3:** Cox proportional hazards model analysis.

Exposure	Non-adjusted	Adjust I	Adjust II	LLR	AIC
Total OS					
Regional_nodes_positive	1.01 (1.00, 1.01) 0.0578	1.01 (1.00, 1.01) 0.0305	1.01 (1.00, 1.01) 0.0924	−2,157.8568	4,319.7137
LNR	1.03 (1.00, 1.06) 0.0720	1.04 (1.01, 1.07) 0.0198	1.04 (1.01, 1.07) 0.0180	−2,157.3056	4,318.6111
LODDS	1.33 (1.20, 1.48) < 0.0001	1.42 (1.28, 1.58) < 0.0001	1.44 (1.29, 1.60) < 0.0001	−2,134.6576	4,273.3151
ELN<10					
Regional_nodes_positive	1.01 (1.00, 1.02) 0.0125	1.01 (1.00, 1.02) 0.0119	1.01 (1.00, 1.02) 0.0407	−829.7002	1,663.4004
LNR	1.03 (1.00, 1.07) 0.0382	1.04 (1.00, 1.07) 0.0255	1.03 (1.00, 1.06) 0.0652	−829.2493	1,662.4986
LODDS	1.55 (1.30, 1.85) < 0.0001	1.65 (1.37, 1.97) < 0.0001	1.51 (1.25, 1.81) < 0.0001	−817.2563	1,638.5126
ELN ≥ 10					
Regional_nodes_positive	1.00 (1.00, 1.01) 0.4372	1.00 (1.00, 1.01) 0.3011	1.00 (0.99, 1.01) 0.4400	−1,326.8883	2,657.7766
LNR	1.01 (0.89, 1.13) 0.9259	1.04 (0.92, 1.17) 0.5466	1.05 (0.93, 1.20) 0.4251	−1,326.7336	2,657.4672
LODDS	1.57 (1.34, 1.85) < 0.0001	1.67 (1.42, 1.97) < 0.0001	1.62 (1.36, 1.93) < 0.0001	−1,314.8367	2,633.6735
Total CSS					
Regional_nodes_positive	1.01 (1.00, 1.01) 0.0883	1.01 (1.00, 1.01) 0.0530	1.01 (1.00, 1.01) 0.1310	−2,078.1839	4,160.3678
LNR	1.01 (0.96, 1.07) 0.6552	1.02 (0.97, 1.07) 0.4247	1.02 (0.98, 1.07) 0.3352	−2,078.0973	4,160.1945
LODDS	1.37 (1.22, 1.55) < 0.0001	1.45 (1.28, 1.63) < 0.0001	1.45 (1.28, 1.64) < 0.0001	−2,060.8769	4,125.7537
ELN < 10					
Regional_nodes_positive	1.01 (0.99, 1.02) 0.2490	1.01 (0.99, 1.02) 0.2409	1.01 (0.99, 1.02) 0.3335	−806.3353	1,616.6706
LNR	1.01 (0.95, 1.07) 0.7048	1.01 (0.96, 1.07) 0.6329	1.01 (0.95, 1.08) 0.6772	−806.1558	1,616.3116
LODDS	1.55 (1.25, 1.91) < 0.0001	1.63 (1.31, 2.02) < 0.0001	1.48 (1.19, 1.84) 0.0004	−798.7815	1,601.5630
ELN ≥ 10					
Regional_nodes_positive	1.01 (1.00, 1.01) 0.2083	1.01 (1.00, 1.01) 0.1379	1.01 (1.00, 1.01) 0.2539	−1,271.5550	2,547.1100
LNR	1.04 (0.92, 1.17) 0.5708	1.06 (0.94, 1.21) 0.3281	1.07 (0.94, 1.23) 0.3184	−1,271.6614	2,547.3227
LODDS	1.71 (1.42, 2.06) < 0.0001	1.79 (1.48, 2.17) < 0.0001	1.72 (1.40, 2.11) < 0.0001	−1,259.8891	2,523.7783

Non-adjusted model adjust for: None; Adjust I model adjust for: Age; Sex; Race;Adjust II model adjust for: Age; Sex; Race; Primary site; Grade; Histology; T stage; N stage; Operation type. ELN, examined lymph node; nPLN, number of positive lymph nodes; LNR, Positive lymph node rate; LODDS, log odds of positive lymph nodes.

In stratified analysis, the LODDS metric demonstrated a significant prognostic value for both lung cancer-specific mortality and all-cause mortality. Patients with LODDS > 0.26 had substantially higher lung cancer-specific mortality in SCC and ADC subgroups (SCC HR = 1.9, 95% CI: 1.1–3.3, *p* = 0.018; ADC HR = 1.7, 95% CI: 1.3–2.4, *p* < 0.001). Furthermore, among patients with N2 staging, LODDS > 0.26 was also associated with a markedly increased mortality risk (HR = 1.9, 95% CI: 1.1–3.4, *p* = 0.025). Consistent with these findings, all-cause mortality was similarly elevated in SCC and ADC patients with LODDS > 0.26 (SCC HR = 1.7, 95% CI: 1.1–2.7, *p* = 0.023; ADC HR = 1.7, 95% CI: 1.3–2.2, *p* < 0.001). Across various histologic subtypes and staging groups, LODDS > 0.26 was closely associated with poorer prognosis, underscoring its potential clinical value in risk stratification and outcome prediction (Table 4).

**Table 4:** Stratified analyses of cohort

	Lung cancer-specific mortality rates HR (95%CI) *P* value		All-cause mortality HR (95%CI) *P* value	
X = LNR categorical	LNR ≤ 0.5	LNR > 0.5	LNR ≤ 0.5	LNR > 0.5
SCC	1.0	1.6 (1.1, 2.5) 0.023	1.0	1.6 (1.2, 2.3) 0.004
ADC	1.0	1.6 (1.3, 2.1) < 0.001	1.0	1.6 (1.2, 1.9) < 0.001
ADSC	1.0	1.6 (1.1, 2.4) 0.009	1.0	1.6 (1.1, 2.2) 0.007
T_stage				
T1	1.0	1.7 (1.1, 2.5) 0.011	1.0	1.7 (1.2, 2.3) 0.002
T2	1.0	1.5 (1.1, 1.9) 0.005	1.0	1.3 (1.1, 1.7) 0.017
T3	1.0	1.7 (1.2, 2.6) 0.005	1.0	1.7 (1.2, 2.4) 0.001
T4	1.0	1.5 (0.8, 2.9) 0.243	1.0	1.5 (0.9, 2.7) 0.141
N_stage				
N1	1.0	1.5 (1.2, 1.8) < 0.001	1.0	1.4 (1.2, 1.7) < 0.001
N2	1.0	2.2 (1.4, 3.4) < 0.001	1.0	2.1 (1.4, 3.0) < 0.001
X = LODDS categorical	LODDS ≤ 0.26	LODDS > 0.26	LODDS ≤ 0.26	LODDS > 0.26
Histology				
SCC	1.0	1.9 (1.1, 3.3) 0.018	1.0	1.7 (1.1, 2.7) 0.023
ADC	1.0	1.7 (1.3, 2.4) < 0.001	1.0	1.7 (1.3, 2.2) < 0.001
ADSC	1.0	1.4 (0.8, 2.3) 0.220	1.0	1.4 (0.9, 2.3) 0.111
T_stage				
T1	1.0	1.3 (0.7, 2.2) 0.403	1.0	1.4 (0.9, 2.2) 0.166
T2	1.0	1.9 (1.4, 2.7) < 0.001	1.0	1.7 (1.2, 2.2) < 0.001
T3	1.0	1.4 (0.8, 2.4) 0.268	1.0	1.5 (1.0, 2.4) 0.078
T4	1.0	1.5 (0.7, 3.5) 0.316	1.0	1.7 (0.8, 3.3) 0.151
N_stage				
N1	1.0	1.6 (1.2, 2.0) < 0.001	1.0	1.5 (1.2, 1.9) < 0.001
N2	1.0	1.9 (1.1, 3.4) 0.025	1.0	2.0 (1.3, 3.3) 0.004
X = nPLN	nPLN ≤ 2	nPLN > 2	nPLN ≤ 2	nPLN > 2
Histology				
SCC	1.0	1.4 (1.1, 1.7) 0.003	1.0	1.4 (1.1, 1.6) < 0.001
ADC	1.0	1.6 (1.3, 1.9) < 0.001	1.0	1.5 (1.2, 1.7) < 0.001
ADSC	1.0	1.6 (1.2, 2.1) < 0.001	1.0	1.6 (1.2, 2.0) < 0.001
T_stage				
T1	1.0	1.8 (1.4, 2.4) < 0.001	1.0	1.8 (1.4, 2.3) < 0.001
T2	1.0	1.4 (1.2, 1.7) < 0.001	1.0	1.3 (1.1, 1.5) < 0.001
T3	1.0	1.3 (1.0, 1.7) 0.061	1.0	1.2 (0.9, 1.5) 0.160
T4	1.0	1.4 (0.9, 2.2) 0.177	1.0	1.5 (1.0, 2.2) 0.048
N_stage				
N1	1.0	1.5 (1.3, 1.7) < 0.001	1.0	1.4 (1.3, 1.6) < 0.001
N2	1.0	2.3 (1.5, 3.5) < 0.001	1.0	2.0 (1.4, 2.8) < 0.001

Note: SCC, squamous cell carcinoma; ADC, adenocarcinoma; nPLN, number of positive lymph nodes; LNR, positive lymph node rate; LODDS, log odds of positive lymph nodes.

### 3.3 The relative validity of LNR, LODDS, and npLN in CSS and OS prediction

**Figure 2:**
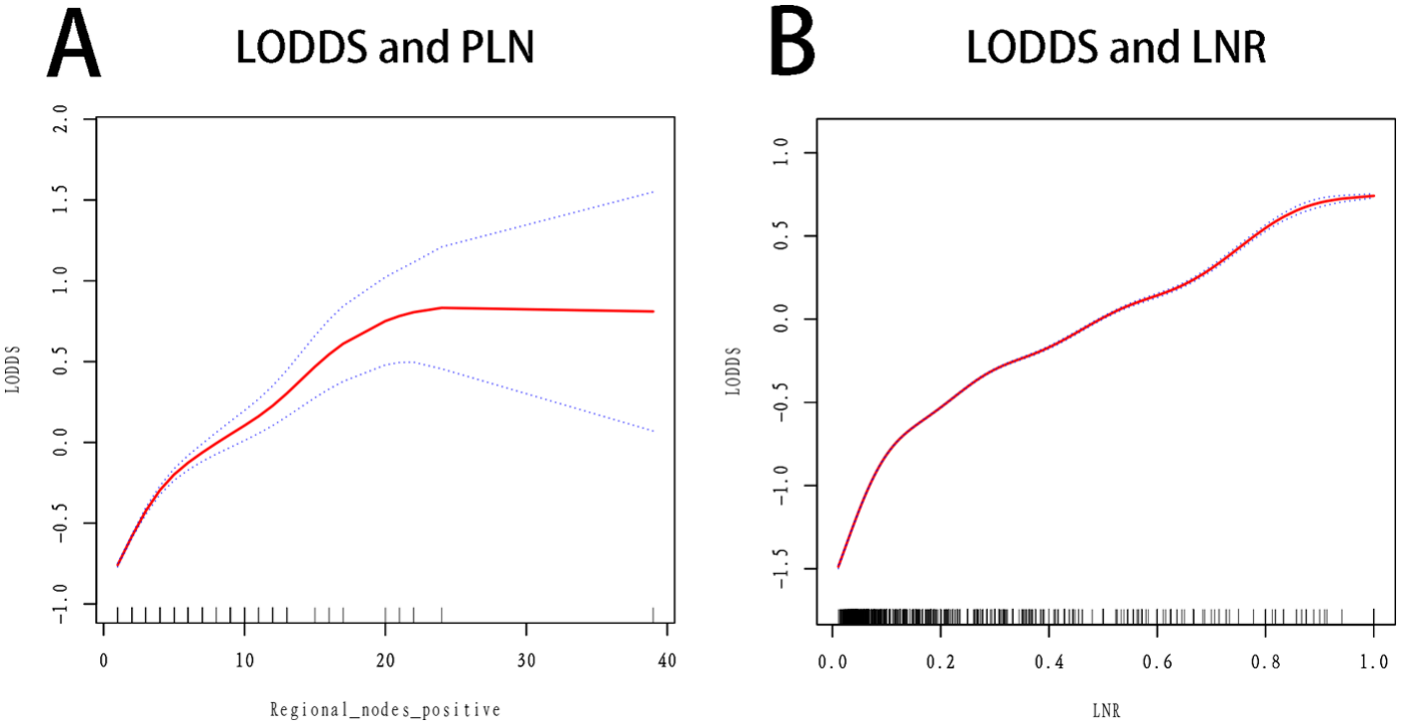
Km curves grouped by LODDS, LNR, nPLN (OS as an indicator of outcome). **(A)** OS by PLN: This Kaplan-Meier curve shows overall survival based on the number of positive lymph nodes (PLN). Patients with PLN ≤ 2 (red line) have a higher survival probability compared to those with PLN > 2 (blue line). The survival difference is statistically significant with *p* < 0.0001. **(B)** OS by LNR: The curve displays overall survival stratified by lymph node ratio (LNR). Patients with LNR ≤ 0.5 (red line) show better survival outcomes than those with LNR > 0.5 (blue line), with a significant survival difference of *p* < 0.0001. C) OS by LODDS: This plot illustrates overall survival categorized by log odds of positive lymph nodes (LODDS). Patients with LODDS ≤ 0.26 (red line) have a significantly better survival probability than those with LODDS > 0.26 (blue line), with *p* < 0.0001.

**Figure 3:**
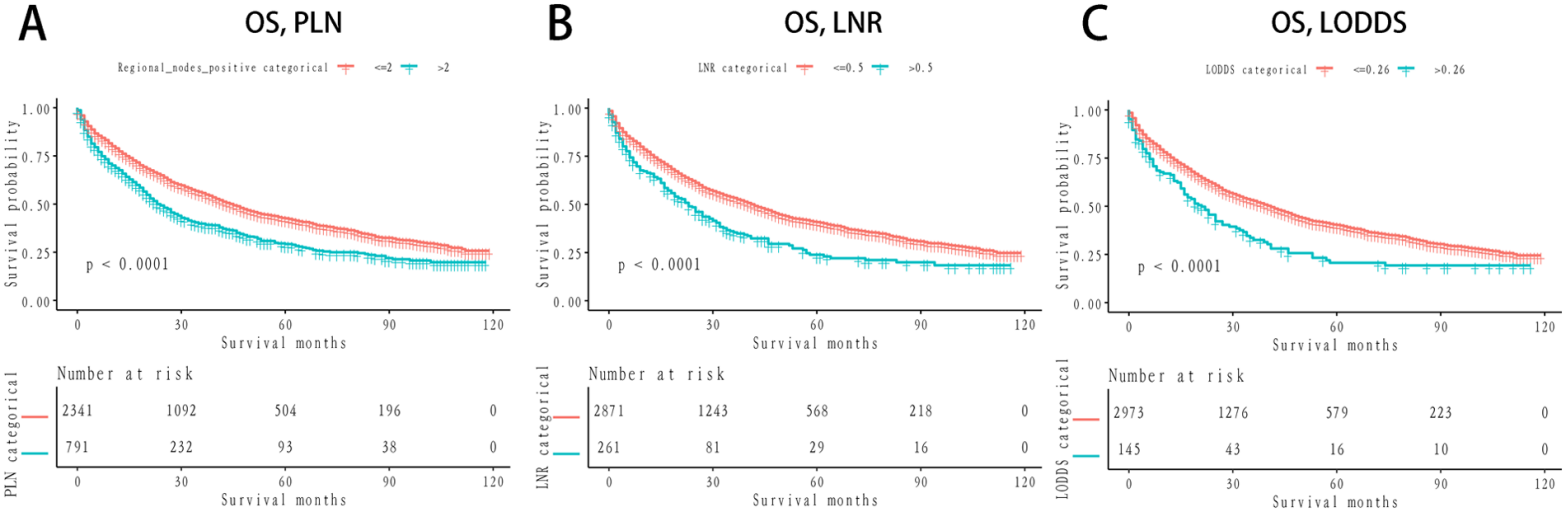
Km curves grouped by LODDS, LNR, nPLN (CSS as an indicator of outcome). **(A)** CSS by PLN: This Kaplan-Meier curve illustrates cancer-specific survival based on the number of positive lymph nodes (PLN). Patients with PLN ≤ 2 (red line) have a higher cancer-specific survival probability compared to those with PLN > 2 (blue line), with a statistically significant difference (*p* < 0.0001). **(B)** CSS by LNR: The curve shows cancer-specific survival stratified by lymph node ratio (LNR). Patients with LNR ≤ 0.5 (red line) display better cancer-specific survival outcomes than those with LNR > 0.5 (blue line), with a significant difference (*p* < 0.0001). **(C)** CSS by LODDS: This plot presents cancer-specific survival categorized by log odds of positive lymph nodes (LODDS). Patients with LODDS ≤ 0.26 (red line) have a significantly better cancer-specific survival probability than those with LODDS > 0.26 (blue line), with *p* < 0.0001.

Among all patients, the LODDS model exhibited superior fit compared to both npLN and LNR (Table 3). Specifically, LODDS demonstrated the most accurate predictive capacity in cohorts with fewer than 10 ELN, characterized by a higher Log Likelihood Ratio (LLR) and a lower Akaike Information Criterion (AIC) which best predicted both OS and CSS. Similarly, in patients with 10 or more examined lymph nodes, LODDS continued to provide superior predictive value for OS and CSS in comparison to both LNR and npLN (Table 3). Higher scores on all three measures—nPLN, LNR, and LODDS—were substantially (*P* < 0.001) related with a worse prognosis, according to Kaplan-Meier analyses (Figures 2, 3).

### 3.4 Feature importance analysis

The XGBoost model was developed to predict OS using a cohort of 2,514 patients in the training set and 618 patients in the validation set. The prognostic relevance of three lymph node-associated features—log odds of positive lymph nodes LODDS, LNR, and the number of positive lymph nodes PLN—was systematically evaluated.

**Figure 4:**
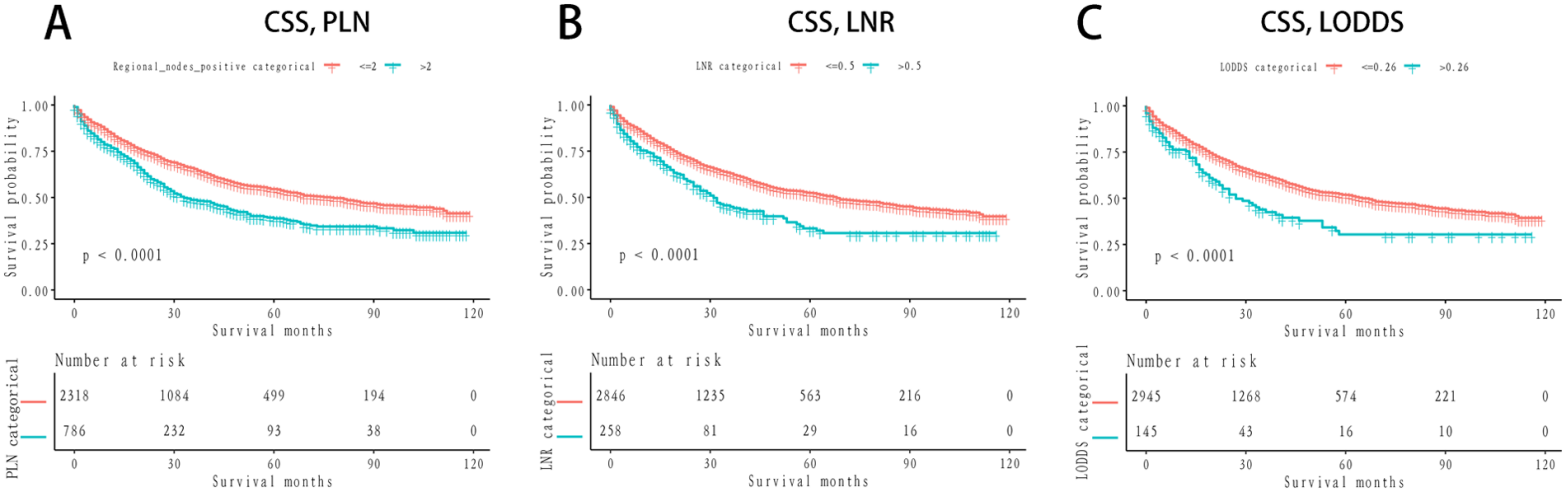
SHAP analysis of feature importance and impact on overall survival prediction using XGBoost. **(A)** SHAP summary plot for the training set. Each dot represents the SHAP value for a single patient, indicating the contribution of the corresponding feature to the predicted overall survival (OS). Features include log odds of positive lymph nodes (LODDS), lymph node ratio (LNR), and the number of positive lymph nodes (nPLN). The horizontal axis shows the SHAP value, with positive values indicating an increased risk of mortality and negative values indicating improved survival. The color gradient represents the feature value, with red indicating higher feature values and blue indicating lower feature values. LODDS exhibits the widest SHAP value distribution, highlighting its dominant role in influencing model predictions. **(B)** Bar chart of mean SHAP values for the training set, showing the average contribution of each feature to the model's predictions. LODDS has the highest mean SHAP value (0.25), followed by LNR, with nPLN contributing the least. This ranking demonstrates the superior importance of LODDS in predicting OS within the training dataset. **(C)** SHAP summary plot for the validation set, using the same feature and SHAP value conventions as in panel **(A)** The validation set results confirm the findings from the training set, with LODDS maintaining the highest impact on model predictions. **(D)** Bar chart of mean SHAP values for the validation set. Similar to the training set, LODDS remains the most important feature, with LNR contributing moderately and nPLN showing minimal importance. This consistency across both datasets underscores the robustness and generalizability of LODDS as a critical predictor of OS.

In both the training and validation sets, LODDS emerged as the most critical feature for OS prediction, exhibiting the highest mean SHAP values (0.25), followed by LNR, while PLN demonstrated the least importance (Figures 4A,C). This hierarchy of feature importance was consistently observed across datasets (Figures 4B,D), underscoring the superior predictive capacity of LODDS.

### 3.5 Feature impact on model predictions

SHAP summary plots further delineated the influence of individual features on model output (Figures 4A,C). Among the features, LODDS displayed the widest distribution of SHAP values, signifying its pronounced impact on survival prediction. Elevated LODDS values were strongly correlated with increased mortality risk, while LNR exhibited a moderate contribution. Conversely, PLN had a negligible effect on model predictions.

These results collectively establish LODDS as the most robust and reliable predictor of OS among the three evaluated features. Its consistent superiority across training and validation cohorts highlights its potential as a pivotal prognostic marker for NSCLC patients undergoing survival risk stratification.

## 4 Discussion

Although the traditional TNM staging system serves as a valuable guide for treatment and prognosis, some early-stage patients who receive standard care still succumb to postoperative recurrence (25). This discrepancy has driven many researchers to explore novel methods to delineate patient heterogeneity within the same TNM stage, aiming to develop a staging system that more accurately forecasts prognosis and directs treatment strategies. LNR has been reported to outperform the conventional N staging system for NSCLC and other malignancies (26). In this study, we sought to further refine the staging paradigm by focusing on the LODDS, which has demonstrated superior prognostic capability compared to TNM-N stage and LNR in breast, colon, and gastric cancers (27–29), though it has yet to be assessed in NSCLC.

The results from the XGBoost model (as shown in Figure 4) clearly demonstrate the superior prognostic value of the log odds of positive lymph nodes compared to the lymph node ratio and the number of positive lymph nodes in predicting overall survival in NSCLC patients. SHAP analysis revealed that log odds of positive lymph nodes consistently exhibited the highest feature importance across both the training and validation datasets, with significantly higher mean SHAP values and broader distributions than the other two lymph node-related parameters. These findings are consistent with previous studies, further validating its prognostic capability. By incorporating both positive and negative lymph nodes, log odds of positive lymph nodes provides a more comprehensive assessment of lymph node burden. Furthermore, the consistent predictive performance in both datasets highlights its potential as a universally applicable prognostic marker. In contrast, lymph node ratio demonstrated moderate utility, while the number of positive lymph nodes contributed minimally, reflecting the limitations of simpler metrics in accurately stratifying survival risk. Collectively, these results support the adoption of log odds of positive lymph nodes as an important complement to, or even a potential replacement for, traditional lymph node evaluation metrics in prognostic models for NSCLC.

Unlike LNR, which is calculated solely from the ratio of positive lymph nodes, LODDS incorporates both positive and negative lymph nodes, thus potentially offering more nuanced prognostic insights for patients with *p*-N0 NSCLC—insights that LNR may not capture. While LNR and LODDS may appear as alternative transformations of similar data, LODDS can provide more detailed prognostic information when LNR values are equivalent. Wang and colleagues posed an intriguing question (30): Is the prognosis of Patient A, who has 4 PLN with 4 lymph nodes removed, the same as Patient B, who has 20 PLN with 20 removed? By extension, we might ask: Does Patient C, with 4 PLN and 0 lymph nodes examined, share the same prognosis as Patient D, with 20 PLN and 0 examined lymph nodes? Intuitively, one would expect Patient A to have a better prognosis than Patient B, and Patient C to fare worse than Patient D. Despite identical LNR values for Patients A and B, and C and D, their respective LODDS values differed (LODDS = 0.95 for Patient A, 1.61 for Patient B, 0.95 for Patient C, and 1.61 for Patient D). Accordingly, our grouping strategy categorized Patients A and B in the LODDS4 group, Patient C in the LODDS2 group, and Patient D in the LODDS1 group. This demonstrates that patients with identical LNR values, particularly zero, can exhibit differing LODDS and thus belong to distinct prognostic groups. Furthermore, an LNR of 0 correlates with N0 status, yet patients classified as N0 may still exhibit varying LODDS and prognoses. The LODDS system, by classifying N0 patients into distinct prognostic categories, holds significant value in shaping treatment strategies. For instance, a patient with N0 disease but a high LODDS should be closely monitored for potential false negatives and subjected to vigilant follow-up.

Staging based on the log odds of positive lymph nodes not only provides superior prognostic predictions for patients with N1 and N2 stages but also differentiates among patients with N0 status. Similar advantages have been documented in studies of breast, colon, and gastric cancers (30). Unlike LNR, which only accounts for positive lymph nodes, LODDS also considers the number of negative lymph nodes, a crucial factor for patients with N0 NSCLC. Previous research indicates that a greater number of examined lymph nodes correlates with higher survival rates, plateauing at approximately 11–16 nodes (31). Since LODDS includes the number of negative lymph nodes, it offers a more comprehensive and effective prognostic indicator compared to LNR.

Extensive research has examined the LODDS, with analyses of 80,000 + breast cancer patients in the SEER database suggesting that LODDS estimates closely align with LNR results (32). A study by the Polish Lung Cancer Group found that LODDS was superior to other classifications involving lymph nodes or LNR for patients with radically resected NSCLC, although it did not specify the number of negative lymph nodes or stratify by the total number of retrieved nodes (33). Our study found that the LODDS model was superior to both npLN and LNR models in Cox regression analysis, suggesting the former's enhanced utility for patients with resected lymph node-positive NSCLC.

Nevertheless, this study has limitations. Lymph node tissue is often fragmented during extraction, which can lead to an overestimation of the number of resected nodes (3, 4, 34). Conversely, difficulties in distinguishing individual lymph nodes from anatomical tissue may result in underestimation. Accurately calculating the number of nodes remains a challenge. The LODDS model, developed to account for both positive and negative lymph nodes, addresses this issue by incorporating a variable that adjusts for the number of nodes collected. Additionally, retrospective studies inherently carry selection bias. Factors not covered by SEER, such as patient comorbidities, performance status, and chemotherapy usage, may also influence our findings. The LODDS model in this study demonstrated potential value in prognostic assessment. However, the lack of external validation may limit its generalizability. Future research should aim to validate the LODDS model using different patient datasets to further confirm its robustness and applicability. Such validation would help ensure the model's utility across diverse populations, thereby improving its clinical applicability.Thus, external validation through large-scale databases is necessary to confirm the predictive accuracy of LODDS for OS and CSS before it can be endorsed for clinical application.

## 5 Conclusions

Compared to the nPLN and LNR staging systems, LODDS demonstrates superior prognostic power for patients with stage I–IIIA NSCLC undergoing lobectomy or pneumonectomy. By integrating both positive and negative lymph node information, LODDS offers a refined risk stratification that is particularly valuable in cases with high lymph node heterogeneity. Clinically, LODDS can serve as a primary tool for identifying high-risk patients, supporting the development of individualized treatment strategies. Incorporating LODDS into routine clinical practice may enhance decision-making processes and improve patient outcomes.

## Data Availability

Publicly available datasets were analyzed in this study. This data can be found here: https://seer.cancer.gov/.

## Supplementary material

Supplementary Figure S1 The best cut-off values for the nPLN and LNR by using X-tile. a) Distribution of PLN: This histogram shows the distribution of patients based on the number of positive lymph nodes (PLN). Most patients have a low PLN value, with a peak near the lower end of the axis. b) X-tile plot for PLN: This plot visualizes the cut-off analysis for PLN, where different shades represent the statistical significance of survival differences between groups. Red indicates a high-risk group, and green indicates a low-risk group. The optimal cut-off point, marked by a white dot, is PLN = 2. c) Risk plot for PLN: This line graph shows the relative risk for different PLN cut-off values. The risk level peaks at PLN = 2, suggesting it as the most significant threshold for prognostic purposes. d) Distribution of LNR: This histogram displays the distribution of patients based on lymph node ratio (LNR). Similar to PLN, most patients have a low LNR, as shown by the concentration at the left end of the axis. e) X-tile plot for LNR: This plot illustrates the optimal cut-off analysis for LNR, with red and green indicating high- and low-risk groups, respectively. The optimal cut-off point, marked by a white dot, is LNR = 0.5, representing the threshold with the most distinct survival separation. f) Risk plot for LNR: This plot displays the relative risk for various LNR cut-off values. The identified cut-off point aligns with a peak in risk at LNR = 0.5, indicating it as a significant threshold for distinguishing prognostic groups.
