# Supplementary figures and images for: Log odds of positive lymph nodes compared to positive lymph node ratio and number of positive lymph nodes in prognostic modeling for patients with NSCLC undergoing lobectomy or total pneumonectomy: a population-based study using Cox regression and XGBoost with SHAP analysis
Source: Front Surg. 2025 Jan 20;11:1530250. doi: 10.3389/fsurg.2024.1530250 (PMC11788378; doi:10.3389/fsurg.2024.1530250)

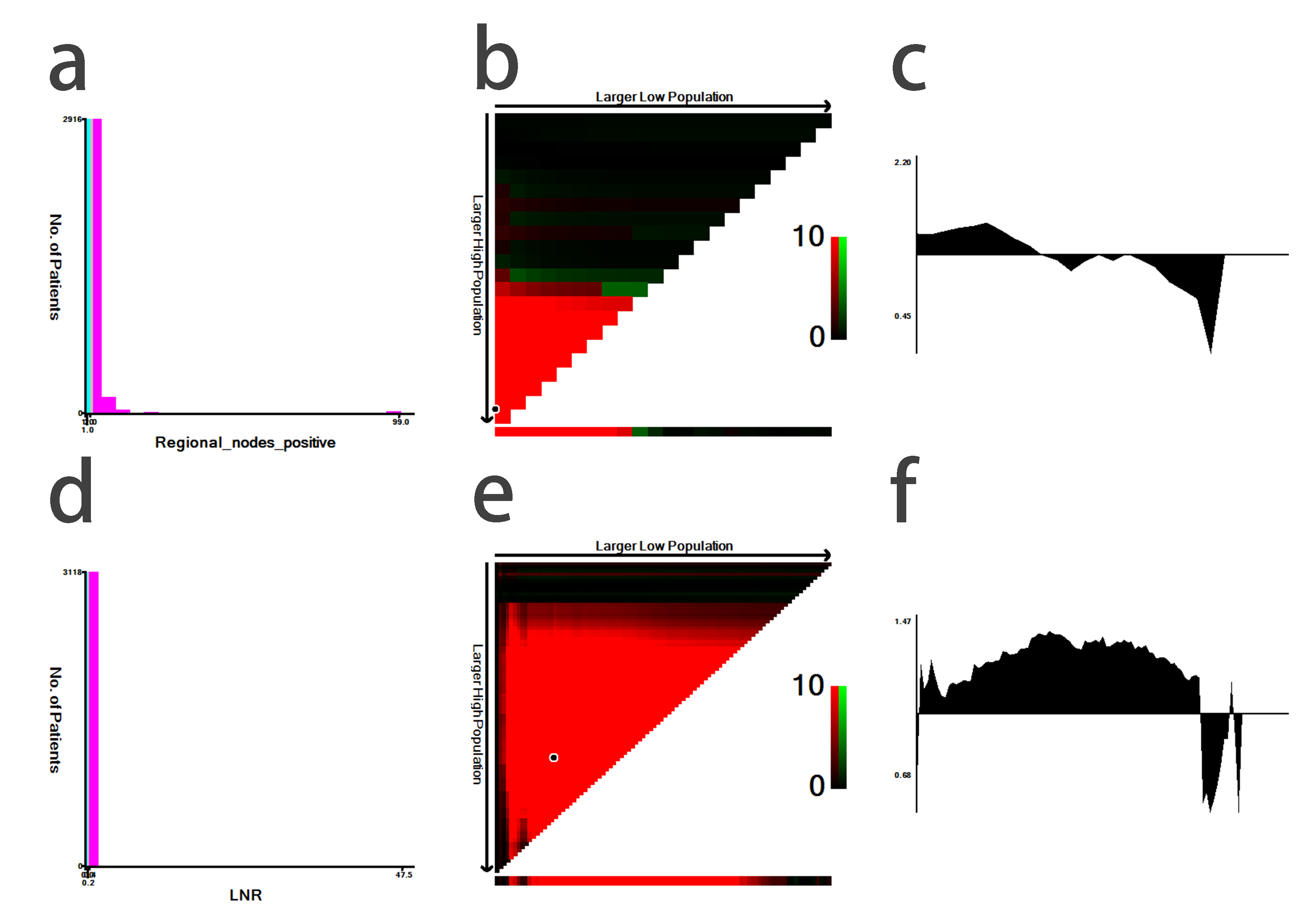

Supplement: Supplementary Figure S1 [file Image1.tif]
